# Association of telomere instability with senescence of porcine cells

**DOI:** 10.1186/1471-2121-13-36

**Published:** 2012-12-15

**Authors:** Guangzhen Ji, Kai Liu, Maja Okuka, Na Liu, Lin Liu

**Affiliations:** 1 State Key Laboratory of Medicinal Chemical Biology; College of Life Sciences, Nankai University, Tianjin, 300071, China; 2 Department of Obstetrics and Gynecology, University of South Florida College of Medicine, Tampa, FL, 33612, USA

**Keywords:** Telomere, Q-FISH, qPCR, Telomere doublets, Telomere dysfunction, Senescence

## Abstract

**Background:**

Telomeres are essential for the maintenance of genomic stability, and telomere dysfunction leads to cellular senescence, carcinogenesis, aging, and age-related diseases in humans. Pigs have become increasingly important large animal models for preclinical tests and study of human diseases, and also may provide xeno-transplantation sources. Thus far, Southern blot analysis has been used to estimate average telomere lengths in pigs. Telomere quantitative fluorescence *in situ* hybridization (Q-FISH), however, can reveal status of individual telomeres in fewer cells, in addition to quantifying relative telomere lengths, and has been commonly used for study of telomere function of mouse and human cells. We attempted to investigate telomere characteristics of porcine cells using telomere Q-FISH method.

**Results:**

The average telomere lengths in porcine cells measured by Q-FISH correlated with those of quantitative real-time PCR method (qPCR) or telomere restriction fragments (TRFs) by Southern blot analysis. Unexpectedly, we found that porcine cells exhibited high incidence of telomere doublets revealed by Q-FISH method, coincided with increased frequency of cellular senescence. Also, telomeres shortened during subculture of various porcine primary cell types. Interestingly, the high frequency of porcine telomere doublets and telomere loss was associated with telomere dysfunction-induced foci (TIFs). The incidence of TIFs, telomere doublets and telomere loss increased with telomere shortening and cellular senescence during subculture.

**Conclusion:**

Q-FISH method using telomere PNA probe is particularly useful for characterization of porcine telomeres. Porcine cells exhibit high frequency of telomere instability and are susceptible to telomere damage and replicative senescence.

## Background

Telomeres consist of (TTAGGG)n repeats and associated proteins at the end of chromosomes in mammalian cells and function in the maintenance of genomic stability to protect the chromosomes from degradation and end-to-end fusion [1,2]. Telomere sequence is conserved among mammals [3,4]. Normal human cells, including the stem cells of renewal tissues, show progressive telomere shortening with cell division until a subset of telomeres reach a critically short length, inducing DNA-damage response and replicative senescence or cell aging [5-7].

Mouse cells may have telomere damage signaling pathways different from those of humans [8]. The observed senescence of mouse cells in culture may not be related to telomere shortening, but rather to changing culture condition, such as excessive oxidative stress [9]. It was proposed that the mouse may not be the best animal model for study of human telomere biology, as fundamental differences exist between human and mouse telomere biology [10]. Other mammals, including dogs, primates, and sheep, have also been compared for telomere biology and function [11-13]. Pigs have been considered as an ideal organ provider for xeno-transplantation and also as appropriate large animal models for preclinical tests and study of human diseases, including cardiovascular disease, diabetes, infectious disease, and cancer, and stem cell therapy [14-17], owing to the similarities in anatomy and physiology between pigs and humans [18]. Also, primary porcine cells have been genetically engineered to induce tumors in size similar to those observed clinically, and may provide a robust cancer model for preclinical studies [19].

Pig telomeres share the conserved TTAGGG sequence of mouse and human telomeres [20,21]. The terminal restriction fragments (TRFs) from pig cells (9–50 kb) are longer than those of human cells (10–20 kb) but shorter than those of laboratory mice (30–200 kb) [11,21]. The major quantitative methods available for telomere measurement include length distribution of TRFs by Southern blot, quantitative fluorescence *in situ* hybridization (Q-FISH) that shows individual telomere lengths of metaphase spreads [22,23], mean telomere length by quantitative PCR (qPCR) [24,25], and PCR of single telomere lengths (STELA) [26]. Pig telomeres have been revealed by fluorescence *in situ* hybridization using human telomere repeat probe (TTAGGG)n [27] and primed *in situ* DNA synthesis (PRINS) [28], but telomere measurement by either method was not quantitative.

TRF measurement by Southern blot was employed to examine telomere lengths in cloned pigs [29,30]. TRFs show distribution of telomeres in smear gels by Southern blots, and only average telomere length is estimated by this approach. However, it is not the average telomere length but rather the shortest telomere that constitutes telomere dysfunction and that becomes a major determinant of the onset of senescence [31,32]. Consistently, chromosome arms carrying the shortest telomeres are the first to be unstable [33]. Telomeres were recently found to resemble fragile sites [34,35]. The shortest telomeres, or fragile telomeres, may reflect DNA-damage response signals in senescent human cells [35,36]. Thus far, quantitative measurement of telomeres at the level of individual chromosomes has not been performed in porcine cells. Moreover, the precise characteristics of pig telomeres and their roles in cellular senescence and immortalization remain elusive. We sought to measure pig telomeres by comparing three methods, Southern blot, Q-FISH, and qPCR, and to characterize pig telomeres in relevance to cellular senescence during subculture of pig primary cells.

## Results

### Telomere lengths shown as TRFs decrease during subculture of pig primary cells

**Figure 1:**
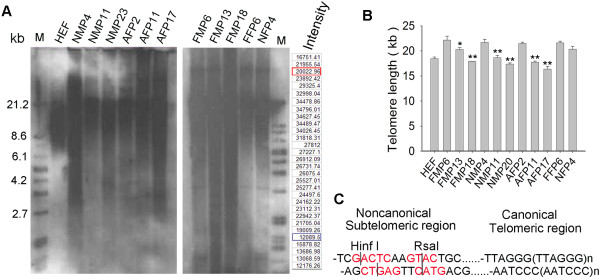
**Telomere length indicated as terminal restriction fragments (TRFs) by Southern blot analysis.** (**A**) Distribution of telomere length as TRFs of various pig cell types. Each lane shows the intensity of TRF from different samples. At last 30 grids were scanned for each sample. Take HEF for example, “Intensity” indicates the scanned intensity of each grid. The Intensity in red and blue rectangle was used as threshold of the upper and lower background, and values above them for calculation of telomere length as detailed in Methods. FF and FM, fetal fibroblast and mesenchymal cells derived from embryonic day 50, respectively; NF and NM, newborn (7 or 8 days after birth) fibroblast and mesenchymal cells, respectively; AF, adult fibroblast from the ear skin of an adult pig, 3–4 months after birth; P, passage. (**B**) Average telomere lengths shown as TRFs in kilobase (kb) pairs of different cell types and changes in telomere lengths during passages. HEF, human embryonic fibroblasts. The data (mean±S.E.) was averaged from three independent experiments. *, p < 0.05; **, p < 0.01, compared to the earliest passage of the same cell types, by ANOVA analysis using Statview software. (**C**) Schematic representation of terminal restriction sites for digestion by enzyme combinations HinfI and RsaI.

Fibroblasts and mesenchymal cells derived from the bone marrow of fetal (embryonic day 50; abbreviated as FF and FM, respectively) and newborn (7 or 8 days old; NF and NM) pigs, as well as fibroblasts from adult pigs (3–4 months of age; AF), during their early passages, did not show significantly different telomere lengths (Figure 1A, B) by Southern blot analysis. Telomere lengths of newborn fibroblasts were slightly shorter than those of fetal fibroblasts during their early passages. Adult pig fibroblasts had telomere lengths similar to those of newborn fibroblasts. Telomere lengths of pig cells, regardless of the age of the animals, were longer than those of human fibroblasts (Figure 1A, B). The telomere lengths also were compared for fetal mesenchymal, newborn mesenchymal, and adult fibroblast cells during subcultures. Telomere lengths of these cells, shown as TRFs, shortened significantly (p < 0.01) from early to late passages (12–16 passage intervals) (Figure 1B). The *Hin*fI/*Rsa*I restriction enzyme mixture was used for genome digestion, and telomeres were digested in the subtelomeric region (Figure 1C), such that TRFs contained lengths of various subtelomeric regions in the samples (Figure 1C). Therefore, measurements of telomeres by Southern blot analysis could show telomere TRF distribution, but the precise status of individual telomeres was not discernible.

### Telomere doublets and signal-free ends increase during subculture of pig cells identified by Q-FISH

**Figure 2:**
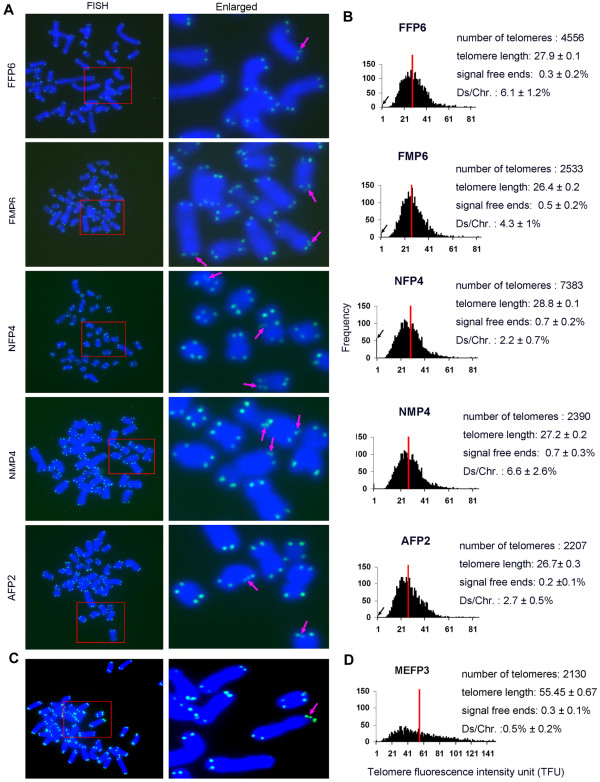
**Telomere length and structure analysis by telomere quantitative fluorescence *in situ *hybridization (Q-FISH).** (**A**) Representative images of telomere Q-FISH in pig cells. Red rectangle, enlarged region shown in the right column. FF and FM, fetal fibroblast and mesenchymal cells derived from fetus at embryonic day 50, respectively; NF and NM, newborn (7 or 8 days after birth) fibroblast and mesenchymal cells, respectively; AF, adult fibroblasts from the ear skin of an adult pig, 3–4 months after birth. P, passages. Enlarged views: Blue, DAPI-stained chromosomes. Green dots, telomeres; Purple arrows, telomere doublets; P, passage. (**B**) Histogram shows distribution of relative telomere length shown as telomere fluorescence intensity unit (TFU) in pig cells. Medium telomere length is shown as mean TFU ± SE. The medium telomere length (red bars) is also shown as mean ± SE in the upper right hand corner. Ds, telomere doublets; Chr, chromosomes. Black arrows on the Y-axis indicate frequency of telomere signal-free ends. (**C**) Representative image of mouse telomeres (green) by Q-FISH. Enlarged view at right. Blue, chromosomes. (**D**) Telomere distribution and length of mouse embryonic fibroblast at passage 3 (MEFP3).

**Figure 3:**
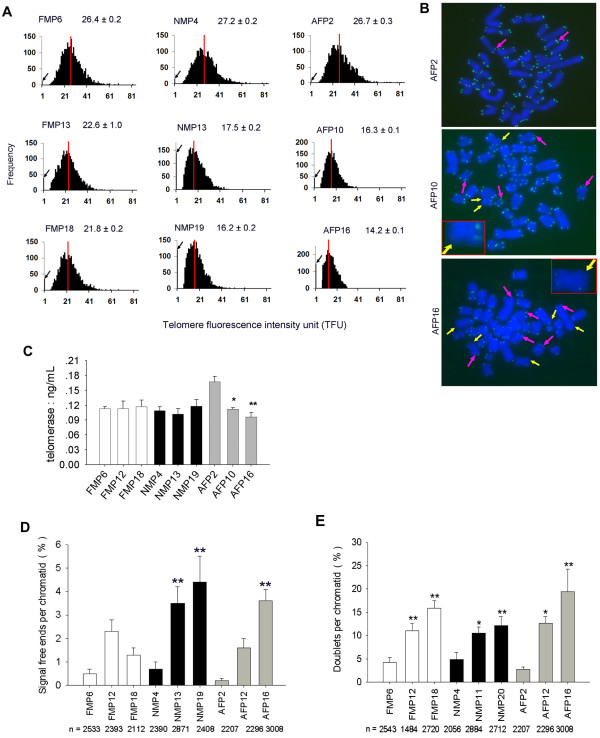
**Telomere length by metaphase Q-FISH analysis of various porcine cell types during subculture.** (**A**) Telomere distribution and length from various pig cells during subculture. The medium telomere length (red bars) is also shown as mean ±SE in the upper right hand corner. Black arrows on the Y-axis indicate frequency of telomere signal-free ends. (**B**) Representative image of adult fibroblasts (AF) during subculture from early to late passage. Purple arrows, telomere doublets; Yellow arrows, telomere signal-free ends. (**C**) Quantification of telomerase activity of various pig cell types by ELLSA. *, p < 0.05; **, p < 0.01, compared to the earliest passage of the same cell type. (**D**) Frequency of telomere signal-free ends in 3 types of pig cells during subculture; n, number of telomeres counted; %, number of signal-free ends per chromatid. (**E**) Percentage of telomere doublets in 3 pig cell types during subculture; %, number of doublets per chromatid. *, p < 0.05; **, p < 0.01, compared to the earliest passage.

To further confirm the TRF results obtained by Southern blot analysis and to analyze individual telomeres, we measured telomere lengths of the pig cell types described above by metaphase telomere Q-FISH using FITC-labeled (CCCTAA) PNA probe. Telomeres, at both terminal and interstitial sites, were found in various pig cell types (Figure 2A). Notably, telomere doublets were typically found at high frequencies at the chromosome ends of all cell types examined (Figure 2A, B). In contrast, only fewer doublets (~0.5%) were observed in mouse embryonic fibroblasts (MEFP3). Consistent with the TRF data, the average relative telomere lengths estimated by Q-FISH did not differ among pig cell types (Figure 2A, B). Not all telomere doublets were clearly separated by the fluorescence signal, so the mean telomere length measured by Q-FISH also contained the signal of the doublets. Fluorescence of one doublet often exhibited a lower signal intensity than the other of the sister doublets. Pig cells during early passages showed a low frequency of telomere signal-free ends (~0.5%), indicative of telomere loss (Figure 2B). Compared to mouse embryonic fibroblasts (Figure 2C, D), pig cells showed shorter telomeres and higher frequency of telomere doublets. Moreover, pig cells, after more passages (FMP18, NMP20, AFP17) during subculture, exhibited telomere shortening, compared to their primary cells (FMP6, NMP4 and AFP2) during the early (7–9) passages (Figure 3A). Interestingly, telomeres shortened less at later passages in these cells (Figure 3A, B, Figure 1B). Telomeres are maintained primarily by active telomerase [1,31]. We determined telomerase activity in various cell types using TE ELISA Kit. Telomerase activity of pig mesenchymal cells and fibroblast cells were detectable (Figure 3C). The telomerase activity of fetal and newborn pig mesenchymal cells did not show noticeable decrease during passages, whereas the activity in adult pig fibroblasts declined during passages. Reduced telomerase activity coincided with evident telomere shortening and loss in adult fibroblasts during passages (Figure 3A, B). Yet, the telomerase activity seemed not to correlate with telomere shortening and loss in newborn mesenchymal cells, and the reason is unclear presently.

During subculture of various pig cell types, frequencies of telomere signal-free ends and doublets increased, accompanied by telomere shortening (Figure 3D, E). High frequency of telomere signal-free ends seemed to coincide with higher incidence of telomere doublets in pig cells.

### Telomere length measurement by quantitative real-time PCR

**Figure 4:**
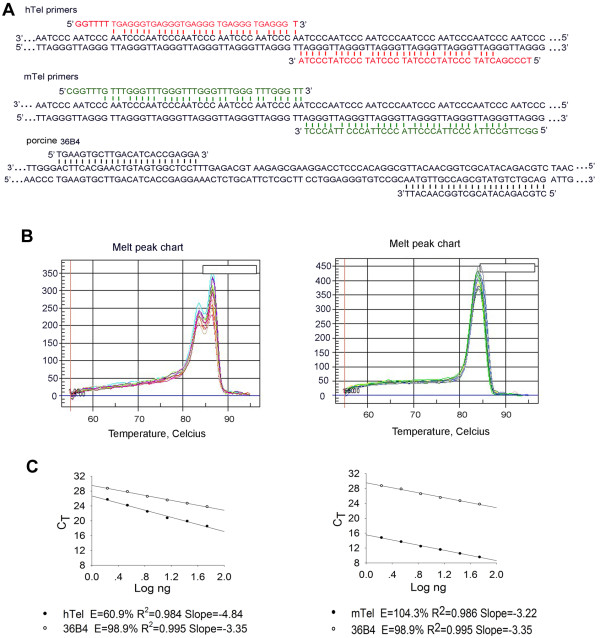
**Optimization of telomere measurement of pig cells by quantitative real-time PCR analysis (qPCR).** (**A**) Telomere primers for pig telomere amplification and 36B4 primers. Telomere primers for human and mouse telomere measurement by qPCR were described previously [23,24]. (**B**) Melting curve charts of human telomere primers and mouse telomere primers for pig telomere analysis, respectively. (**C**) Standard curves for pig telomere amplification, using human telomere primers and porcine 36B4, mouse telomere primers and porcine 36B4, respectively. Telomeres and 36B4 for standard curves were derived by serial dilution of a known quantity of genomic DNA isolated from spleen cells. hTel, primers for human telomere sequence amplification; mTel, primers for mouse telomere sequence amplification; E, amplification efficiency; CT, threshold cycle. Solid circle, telomeres; Hollow circle, 36B4 control.

An additional method was required to further confirm the data obtained by Q-FISH. We showed that both qPCR and Q-FISH methods complement each other for telomere measurement in mouse cells [37]. Initial experiments were designed to determine whether human and/or mouse telomere primers could efficiently amplify pig telomeres by qPCR (Figure 4A). Pig 36B4 was used as an internal control. Samples for both mouse and human telomeres and 36B4 amplification were run under the same conditions. We found that primers for mouse telomeres (mTel) were suitable for pig telomere amplification, based on amplification efficiency and melting curves (Figure 4A, B), whereas pig telomere amplification efficiency using human telomere primers was only 60.9% (Figure 4C). In addition, 35 ng of DNA was suitable for amplification of both pig telomeres and 36B4 (Figure 4C).

**Figure 5:**
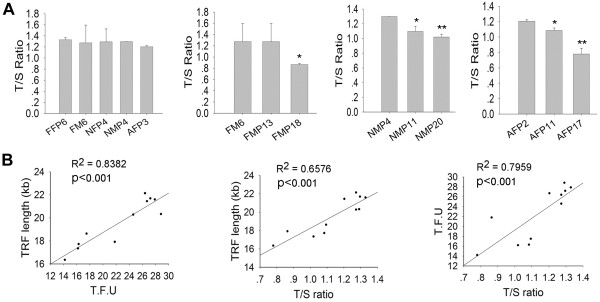
**Telomere measurements of pig cells shown as a T/S ratio by qPCR and its correlation with TRFs, and TFU by Q-FISH.** (**A**) Average relative telomere length shown as T/S ratios of various cell pig cells and during passage by qPCR method. T, telomere; S, 36B4 single-copy gene. P, passage. *, p < 0.05; **, p < 0.01, compared to the earliest passage. (**B**) Correlation of telomere length between TFU by Q-FISH, T/S ratio by qPCR, and TRFs (kb) by Southern blot.

By qPCR analysis, telomeres from five cell types showed similar lengths, with relatively shorter telomeres in adult fibroblasts (Figure 5A), consistent with the results obtained by Southern and Q-FISH methods. Further, telomeres shortented in pig fetal mesenchymal cells, newborn mesenchymal cells, and adult fibroblasts from early to late passages (p<0.01) (Figure 5A). Regression analysis indicated that the average telomere lengths measured by Southern, Q-FISH, and qPCR were highly (P<0.001) correlated with each other, showing high R^2^ between Q-FISH and Southern, qPCR and Southern, and Q-FISH and qPCR as 0.84, 0.66 and 0.80, respectively (Figure 5B).

### Increased telomere dysfunction with accumulation of senescent cells

**Figure 6:**
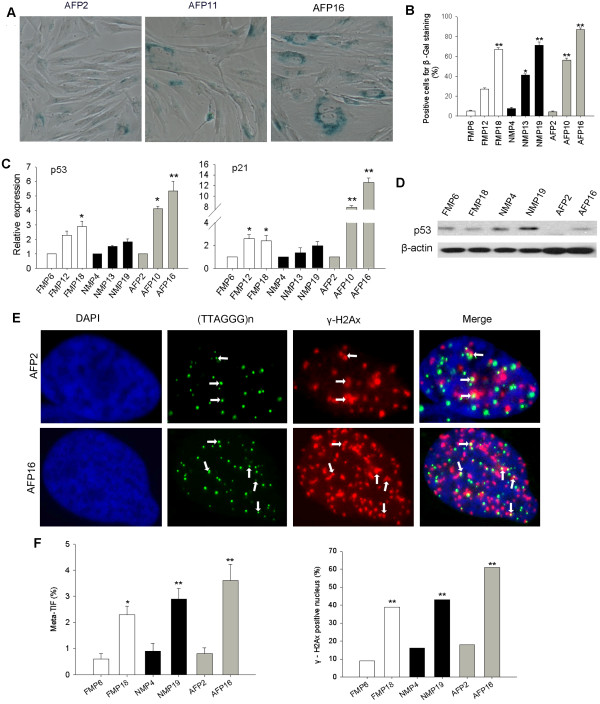
**Telomere dysfunction is associated with cellular senescence in pig cells.** (**A**) Morphology of adult fibroblasts (AF) during subculture by β-galactosidase staining. P, passage. Senescent cells are stained blue. (**B**) Quantification of senescent cells positive for β-galactosidase staining. (**C**) Relative expression levels by qPCR of the senescence-related genes, *p53* and *p21*, in 3 types of pig cells during subculture. *, p < 0.05; **, p < 0.001, compared to the primary cells at the earliest passage. (**D**) P53 protein levels in different cell lines from early to late passages by immuno-blot analysis. β-actin served as loading control. (**E**) Representative images showing TIFs as DNA damage foci (white arrows) indicated by γ-H2AX foci at telomeres by IF-FISH. Nuclei, blue; Telomere, green; γ-H2AX, red. (**F**) Percentage of TIFs and γ-H2AX-positive nucleus in various pig cell types. In all, 100 cells were counted for each cell line. *, p < 0.05; **, p < 0.01, compared to the early passage of the same cell type.

We speculated that telomere shortening and dysfunction are associated with cell senescence in pig cells during passaging. Cellular senescence was evaluated by standard β-galactosidase (β-Gal) assay (Figure 6A). Indeed, the incidence of β-Gal-positive cells increased remarkably with increasing passages, regardless of the cell types (Figure 6B). Senescence can be triggered by up-regulation of *p53* and *p21*[38], and *p53*-dependent senescence responds to dysfunctional telomeres [38,39]. Consistently, expression levels of *p53* and *p21* increased during subculture of pig cells (Figure 6C), in association with telomere shortening. Changes in p53 protein levels were verified by Western blot (Figure 6D). γ-H2AX foci colocalized with telomeres, as indicative of telomere dysfunction-induced foci (TIFs) [40], were analyzed for various cell types. The percentage of TIFs by IF-FISH and of cells with DNA damage increased significantly at later passages (Figure 6E, F).

## Discussion

We compared three methods for telomere measurement in pig cells. Using telomere Q-FISH method [23,41], interestingly, we found that pig cells show high incidence of telomere doublets. Pig lymphocytes at metaphase were analyzed by FISH with telomeric DNA probes to identify telomeres at both terminal and interstitial sites, but the method was not sensitive enough to reveal telomere doublets [27]. Telomeres in interphase nuclei of pig ovarian follicles were also measured by FISH with telomeric DNA probes [42], but the overlapping of telomere signals in interphase nuclei might overestimate the telomere signals. We show here that the Q-FISH method using telomeric PNA probe is very useful in determining both the lengths and the status of pig telomeres.

Telomere qPCR yields T/S ratio and is a time-saving method for relative quantitative measurement of telomeres. In addition, the method only requires a small amount of sample [24,25]. We slightly modified the telomere amplification conditions by amplification of both telomere and 36B4 primers in one set of reaction. We found a high correlation of T/S ratio measured by qPCR with telomere length measured by Q-FISH in mouse stem cells [37]. The T/S ratio determined by qPCR contained lengths of both terminal and interstitial telomere sequences. Interestingly, telomeric primers for mouse, but not human telomeres, are suitable for pig telomere measurement by qPCR. The reason is unclear, but might relate to higher amplification efficiency for longer telomeres of pig cells. Together, telomere lengths measured by three methods showed strong correlation, proving effective measurement of relative telomere lengths by each method. Telomere structure, loss, and doublets, however, can only be revealed by Q-FISH method.

Telomere lengths of pig skin fibroblasts at early passages show no appreciable differences among individuals. Yet, telomeres shorten significantly during subculture of various pig cell types. Also, human fibroblasts undergo replicative senescence, accompanied by accumulation of short telomeres and loss of telomeric sequences [6,38,43], and the accumulated telomeric loss accelerates senescence [44]. Up-regulation of *p53* and *p53-*induced gene, *p21*, is associated with cell senescence [38,45,46]. Likewise, porcine cells show increased expression levels of *p53* and *p21*, along with telomere shorting and cell senescence. Telomere dysfunction, accompanied by cell senescence, has been shown to appear early in human cells [38,47]. It appears that pig cells undergo senescence in a manner similar to human cells.

Notably, pig cells exhibit telomere doublets at a high incidence. Primary pig cell lines showed approximately 5% doublets, similar to human cells [48]. Telomere doublets often refer to more than one telomeric signal at a single chromatid end [49]. Telomere doublets may indicate fragile telomeres, following damages to telomeres [49-52]. Telomere doublets are indicative of genome instability. Telomere doublets are found in both normal and mutant human cells [48], and also associated with fragile telomeres and cellular senescence [34,53]. Increased frequency of telomere doublets during subculture of pig cells also coincides with telomere loss and dysfunction, in association with cellular senescence. Both telomere doublets and telomere loss represent telomere instability, and telomere doublets may lead to telomere shortening and loss under stress conditions. Telomere doublets may provide a marker for cell senescence.

## Conclusion

Porcine cells exhibit high frequency of telomere doublets and are susceptible to telomere damage and replicative senescence. Telomere biology of pigs appears to be similar to that of humans. Investigation of telomere dysfunction and senescence using pig cells may complement studies of human biology and medicine.

## Methods

### Cell culture

The use of the animals for this research was approved by the Institutional Animal Care and Use Committee at Nankai University. Porcine (Yorkshire) fibroblasts from fetal (embryonic day 50), newborn (7 or 8 days after birth), and adult (3–4 months) pigs were isolated by a standard tissue-attachment method. The upper layer of pig ear skin was removed, and the remaining tissue was minced into 1 × 1-mm^3^ pieces, placed to culture dishes, and incubated in Dulbecco’s Modified Eagle Medium (DMEM)(Gibico, USA), containing 10% fetal bovine serum (FBS)(Thermo, USA). Gelatinous bone marrow tissues from fetal femurs and tibias, at embryonic day 50, as well as from newborn piglets were extracted under sterile conditions. Bones were rinsed with DMEM after both ends were cut. The recovered cells were centrifuged, resuspended, and plated in culture dishes. The culture medium containing 15% FBS, was changed every other day, and cells passaged every 3 or 4 days when the cells reached 90% confluence. Human fibroblasts (HEF) were provided by Z. Liu at Sun Yat-Sen University.

### Chromosome spreads and telomere Q-FISH

Cells were incubated with 0.2–0.3 μg/mL nocodazole (Sigma, St. Louis, MO, USA) for 3 h to enrich the cells in metaphase. Chromosome spreads for different cell types were made using a routine method. Metaphase-enriched cells were subjected to hypotonic treatment in a 75 mM KCl solution, fixed with methanol:glacial acetic acid (3:1), and spread onto clean slides. Telomere FISH and quantification were performed as described previously [22,23,41], except that a fluorescein isothiocyanate (FITC)-labeled (CCCTAA) peptide nucleic acid (PNA) probe was used in this study. Telomeres were denatured at 80°C for 3 min and hybridized with telomere PNA probe (0.5 μg/mL) (Panagene, Daejeon, Korea). Chromosomes were stained with 0.5 μg/mL 4',6-diamidino-2-phenylindole (DAPI). Fluorescence signals from chromosomes and telomeres were digitally imaged using a Zeiss microscope with FITC/DAPI filter sets, in combination with AxioCam and AxioVision software 4.6. For quantitative measurement of telomere length, telomere fluorescence intensity was integrated using the TFL-TELO program (a gift kindly provided by P. Lansdorp, Terry Fox Laboratory, Vancouver, Canada). More than 15 metaphase spreads were examined for each cell type.

### Telomere measurement by quantitative real-time PCR

A modified qPCR method by amplification of both telomeres and the single-copy gene (36B4) within one set of reactions proves to be effective for measurement of telomere length in early embryonic and pluripotent stem cells in mice [37,54]. DNA samples were extracted from various cell types using the DNeasy Blood & Tissue Kit (Qiagen, Valencia, CA, USA). The average telomere length was measured from total genomic DNA using a real-time PCR assay, as previously described [24,25,37], with slight modifications for measurement of pig telomeres using human or mouse telomere primers: human telomeric primers (5^′^-3^′^): (hTel) Forward, GGTTTTTGAGGGTGAGGGTGAGGGTGAGGGTGAGGGT; hTel Reverse, TCCCGACTATCCCTATCCCTATCCCTATCCCTATCCCTA; murine telomere (mTel) Forward, CGGTTTGTTTGGGTTTGGGTTTGGGTTTGGGTTTGGGTT; mTel Reverse, GGCTTGCCTTACCCTTACCCTTACCCTTACCCTTACCCT. Primers for the reference control gene (pig 36B4 single-copy gene): forward, TGAAGTGCTTGACATCACCGAGGA; reverse, CTGCAGACATACGCTGGCAACATT. The PCR amplification system for telomeres included a SYBR Green Mix of 12.5 μL, 400 nM forward and reverse telomere primers. The reaction system for the 36B4 gene contained the same amount of SYBR Green mix as telomeres, 400 nM forward primer and 640 nM reverse primer. Samples with equal amounts (35 ng) of DNA were added to 2 adjacent wells. A sufficient quantity of double-distilled water was added to each well to yield a final volume of 25 μL. Both telomeres and the 36B4 gene were amplified under the same conditions. For each PCR reaction, a standard curve was made by serial dilution of known amounts of DNA. All PCR reactions were performed using iCycler iQ real-time PCR detection system (Bio-Rad, Hercules, CA, USA). Pig spleen DNA diluted from 110 to 1.7 ng was amplified for the standard curve. The telomere (T) signal was normalized to the signal from the single-copy (S) gene to generate a T/S ratio indicative of relative telomere length. Equal amounts of DNA (35 ng) were used for each reaction, with at least 3 replicates for each specimen.

### Terminal restriction fragment (TRF) assay

Genomic DNA from the different samples was prepared as above and DNA was dissolved in nuclease-free water. The DNA quality was assayed using a spectrophotometer, and the ratio of 260 to 280 was between 1.8 and 2.1. The mean telomere length was determined by mean TRF length analysis using TeloTAGGG Telomere Length Assay Kit (Roche, Mannheim, Germany), according to the protocol provided by the manufacturer, with slight modifications. Each DNA sample (3 μg) was digested with *Hin*fI and *Rsa*I overnight. The digested DNA was separated by agarose gel (0.8%) electrophoresis at 5 V/cm in 1× TBE buffer for 4 h. Gels were denatured, neutralized, and transferred to positively charged nylon membranes (Amersham, Oakville, ON, Canada) overnight. The membranes were prehybridized in DIG Easy Hyb (Roche) at 42°C for 45 min, and then hybridized in DIG Easy Hyb containing the telomere probe at 42°C for 3 h. The membranes were washed, blocked, and incubated with Anti-DIG-AP working solution and detection buffer. They were then subjected to chemiluminescence detection according to the manufacturer’s instructions and exposed to X- ray film for 1–5 min. The mean telomere length was calculated using the following formula: *TRF* = *Σ*(*OD*_*i*_)/*Σ*(*OD*_*i*_/*L*_*i*_), where OD_i_ is the chemiluminescent signal and OD_i_/L_i_ is the length of the TRF at position i.

### Gene expression analysis by quantitative real-time PCR

Total RNA was isolated from porcine cells by using an RNeasy mini kit (Qiagen). RNA (2 μg) was subjected to cDNA synthesis using M-MLV Reverse Transcriptase (Invitrogen, Grand Island, NY, USA). Real-time quantitative PCR reactions were set up in duplicate with the SYBR Green Master (TOYOBO) and run on the iCycler iQ5 2.0 Standard Edition Optical System (Bio-Rad). Each sample was repeated 3 times and analyzed with *β-actin* as the internal control. Most primers were designed using the IDT DNA website (http://www.idtdna.com/Home/Home.aspx) as follows: *p21* forward, 5^′^ACCATGTGGACCTGTTGCTGT3^′^, and reverse, 5^′^AGAAATCTGTCATGCTGGTCTGCC3^′^; *p53* forward, 5′GGAACAGCTTTGAGGTGCGTGTTT3^′^, and reverse, 5^′^AATACTCGCCATCCAGTGGCTTCT3^′^; *β-actin* forward, 5^′^TGCGGCATCCACGAAACTAC3^′^, and reverse, 5^′^TTCTGCATCCTGTCGGCGAT3^′^.

### Senescence β-galactosidase staining

Cells were stained using a senescence kit (Beyotime, C0602, Shanghai, China), according to the manufacturer’s instructions. Cells were fixed with formalin for 15 min at room temperature, washed with PBS 3 times, and incubated at 37°C with β-galactosidase working solution overnight. Samples were mounted, and imaged under a microscope (ECLITSE-TS100, Nikon, Japan). At least 5 pictures were randomly captured for each sample. More than 10,000 cells were counted for quantification of senescent cells.

### Western blot analysis

The P53 protein expression levels were analyzed for cultured pig cells at early and late passages. Primary antibody against P53 (sc-126, Santa Cruz) was used for Western blotting and β-actin (P30002, abmart) served as loading controls. Cells were lysed in lysis buffer plus 1mM PMSF. Protein concentration was quantified by Pierce® BCA Protein Assay Kit (23227 Thermo). The protein samples (20 μg) were separated on 10% SDS-polyacrylamide gels and transferred to a PVDF membrane (0.2 μm Millipore ISEQ00010). After blocking with TBST buffer containing 5% nonfat dry milk, the membrane was incubated with the primary antibodies. After washing and incubation with secondary antibody (ECL anti-rabbit/mouse IgG, NA934/NA931 GE Healthcare), the proteins were detected using chemiluminescent HRP substrate reagent (WBKLS0100, Millipore).

### Telomerase activity assay

Telomerase activity of pig cells was quantified using TE ELISA Kit (CSB-E06793p, CUSABIO, China) according to the manufacturer’s protocol. 1×10^6^ cells in 300 μl PBS were used for telomerase quantification, and 100 μl Standard, Blank, or Sample added per well, covered with the adhesive strip, and incubated for 2 h at 37°C. Biotin-antibody working solution (100 μl) was added to each well and incubated for 1 h at 37°C. After washing for three times, 100 μl HRP-avidin working solution was added to each well, covered with a new adhesive strip, and incubated for 1 h at 37°C. After washing, 90 μl TMB Substrate was added to each well, and incubated for 15–30 min at 37°C. The optical density of each well was determined using a microplate reader set to 450 nm.

### Telomere dysfunction induced foci (TIFs) by immunofluorescence (IF)-telomere FISH (IF-FISH)

IF-FISH was performed as described previously [34,40]. Briefly, cells were grown on gelatin-treated cover slips and fixed with 2% paraformaldehyde for 10 min at room temperature. The cells were washed with a blocking solution (1 mg/mL bovine serum albumin, 3% goat serum, 0.1% Triton X 100 and 1 mM EDTA pH 8.0) and incubated with anti-γH2AX (Upstate, 05–636, CA) in blocking solution. The secondary antibody against mouse IgG was labeled with Alexa Flour 594 (Invitrogen). Cells were fixed in 2% paraformaldehyde for 5 min, and FISH was performed using a FITC-(CCCTAA)_3_ PNA telomere probe (Panagene), as described above. DNA was counterstained with 0.5 μg/L DAPI in Vectashield mounting medium (Vector Laboratories, Burlingame, CA). Fluorescence was detected and imaged using a Zeiss Imager Z1 microscope equipped with an epifluorescence source and lenses.

### Statistical analysis

Percentages were transformed using arcsine transformation. Transformed percentage data and other numbers were analyzed by analysis of variance (ANOVA), and means were compared by Fisher’s protected least-significant difference (PLSD) test using StatView software from SAS Institute Inc. (Cary, NC). Linear relationships and regression analysis were performed using SigmaPlot 8.0 (Systat Software, San Jose, CA, USA). Significant differences were defined as p < 0.05, 0.01, or lower.

## Competing interest

The authors declare that they have no competing interests.

## Authors’ contributions

GJ, KL, MO, NL performed experiments and data analysis. GJ designed the experiments and wrote the manuscript; LL designed and advised the experiments, and revised manuscripts. All authors read and approved the manuscript.
